# Experiences of patients living with HIV and AIDS on antiretroviral therapy in Accra, Ghana

**DOI:** 10.4102/curationis.v47i1.2444

**Published:** 2024-04-16

**Authors:** Joana Agyeman-Yeboah, Esmeralda J. Ricks, Margaret Williams, Portia J. Jordan

**Affiliations:** 1 Department of Nursing, International Maritime Hospital, Tema, Ghana; 2 Department of Nursing, Knutsford University College, Accra, Ghana; 3 Department of Nursing Science, Nelson Mandela University, Port Elizabeth, South Africa; 4 Department of Nursing and Midwifery, Stellenbosch University, Cape Town, South Africa

**Keywords:** acquired immunodeficiency syndrome, antiretroviral therapy, discrimination, human immunodeficiency virus, patients living with HIV and AIDS, stigmatisation

## Abstract

**Background:**

The human immunodeficiency virus and acquired immunodeficiency syndrome (HIV and AIDS) pandemic has greatly affected Africa, particularly Ghana. The pandemic remains a public health concern, particularly in terms of accessing essential medication and improving quality of life for people living with the disease.

**Objectives:**

This study aimed to explore and describe the experiences of persons diagnosed and living with HIV who are on antiretroviral therapy.

**Method:**

A qualitative, exploratory, descriptive, and contextual design was used. The research population included persons diagnosed with HIV who were receiving antiretroviral therapy at three public hospitals in Ghana. Data saturation was achieved after conducting 15 semi-structured interviews. Creswell’s six steps of data analysis were used to analyse the data, which resulted in the emergence of one main theme and six sub-themes.

**Results:**

The main theme identified by the researchers highlighted the participants’ diverse experiences of being diagnosed and living with HIV. It was found that the study participants expressed shock, disbelief, surprise, and fear of death after being diagnosed with HIV. The participants also experienced stigmatisation, discrimination, and rejection.

**Conclusion:**

There is a need for further research on the extent of discrimination and stigmatisation and the effect on optimal adherence to antiretroviral therapy. Continuous public education on HIV is required to limit the extent of discrimination and stigmatisation.

**Contribution:**

The study has highlighted the various emotions related to stigma and discrimination expressed by persons living with HIV (PLHIV). The findings will guide policy on eliminating discrimination and stigmatisation for people living with HIV.

## Introduction

Human immunodeficiency virus and acquired immunodeficiency syndrome (HIV and AIDS) is a global pandemic that continues to be a serious public health concern. According to the Joint United Nations Programme on HIV/AIDS (UNAIDS 2021a), by the end of 2020, there were 37.6 million people estimated to be living with HIV, and approximately 690 000 people died from AIDS-related diseases in the same year. Nearly 34.7 million have died from AIDS-related illnesses since the start of the epidemic. However, the burden of the epidemic varies considerably between countries and regions, with sub-Saharan Africa remaining the most severely affected area. The literature reviewed provided evidence that as the number of people living with HIV increases, so do their experiences of rejection and discrimination among friends, family members, and employers. It is undeniable that perceived stigma and fear of judgement persist among persons living with HIV (PLHIV) (Stangl et al. 2019). A systematic review of qualitative studies on Experiences and Attitudes of People with HIV/AIDS with Articles from North America, South America, Central America, Europe, and Africa reported that because of feelings such as dissatisfaction, grief, fear, despair, a lack of awareness, and suffering, the moment of diagnosis is crucial. High importance is placed on social support, which has been shown to enhance quality of life. People with HIV/AIDS experience various forms of stigma, including self-stigma, social stigma, and stigma from healthcare professionals (Arias-Colmenero et al. 2020).

Stigma and discrimination in the workplace marginalise people, pushing those with HIV into poverty. According to the International Labour Organization, myths and misconceptions about HIV and AIDS fuel stigma and discrimination in the workplace (UNAIDS 2021b). Concerns about the fear of being stigmatised influence an individual’s decision to get tested and lead to reluctance to ask for help and access healthcare. Equally, it leads to withholding information about their status from family members, friends, and care providers (Better Health Channel 2021). HIV and AIDS has been accepted globally as a chronic disease, which means that each person infected with HIV will eventually require treatment. Since 2016, the World Health Organization (WHO) has recommended ‘Treat All’: that all people living with HIV be provided with ART initiation including offering antiretroviral therapy (ART) on the same day as diagnosis to those who are ready to start treatment (WHO 2022).

The Joint United Nations Programme on HIV/AIDS proposed a 95-95-95 objective as a way to accelerate HIV treatment. As a result, 95% of HIV-positive individuals need to be aware of their condition. Then the 95% of HIV-positive individuals who are aware of their status must begin antiretroviral treatment, with the goal being for 95% of those receiving treatment to have suppressed viral levels by 2025 (UNAIDS 2017). The 95-95-95 target’s accomplishment significantly hinges on strict adherence to the ART programme. Taking one’s medication precisely as directed has been defined as adhering to the regimen. This suggests that the correct dosage should be given on time and under appropriate circumstances. Evidence shows that non-adherence to ART is common globally, including Africa, and specifically Ghana. Studies by Abadiga et al. (2020), Aye, Puckpinyo and Peltzer (2017), Semvua et al. (2017), and Yu et al. (2018) determined that North Tanzania had ART adherence percentages of 71%, 84%, and 85%, respectively. Ghana’s ART adherence is lower than that of most other countries, at 62% – 73%, and Nigeria’s is between 54% and 62%, according to Obirikorang et al. (2013). Research findings suggest that a fixed-dose combination reduces pill burden and improves adherence (Ebrahim & Mazanderani 2013). The experiences of PLHIV on antiretrovirals (ARVs) are important in terms of ascertaining how the daily medications required for survival influence their lives.

Most researchers in Ghana have focused on the experiences of people living with HIV (Dako-Gyeke, Dako-Gyeke & Asampong 2015; Doat, Navab & Hoseini 2020; Owusu 2020, 2022) and have recorded findings such as a lack of family support, suicidal thoughts, discrimination, and marriage breakdowns, among others. What is unclear is whether the experiences differ from the burden of taking the ARVs and adhering to the therapy to enhance improved health. Acknowledging the experiences of PLHIV receiving ART to offer baseline data for policy recommendations is necessary. The research question underpinning this study was: How do people diagnosed with HIV/AIDS experience living the rest of their lives on antiretroviral therapy? Thus, this study aimed to investigate and report the experiences of HIV/AIDS patients receiving ART medication at public hospitals in Ghana. In this study, patients receiving ART at three public hospitals in Ghana provided qualitative data, examining their adherence to the therapy.

## Research methods and design

A qualitative strategy was employed to conduct this study, which was explorative, descriptive, and contextual. This design allowed the researcher to explore and describe the experiences of people living with HIV/AIDS and who are on ART (Grove, Burns & Gray 2013). The study sought to explore and describe how these people manage their daily lives while having to take medication for the rest of their lives. This study was conducted at three public hospitals, the major treatment centres in Accra, Ghana that provide care and treatment to patients diagnosed with HIV/AIDS. The research population consisted of all patients diagnosed with HIV who were receiving treatment at any of the three hospitals. Purposive sampling was employed to select the study participants, ensuring equity and fairness maintenance (Creswell 2014). This sampling technique allowed the researcher to intentionally recruit participants who met the inclusion criteria for the study. The gatekeeper, in this case, the in-charge nurse of HIV clinics, contacted the participants on behalf of the researchers. The gatekeeper was briefed about the inclusion criteria used to recruit participants for the study. The gatekeeper allowed all those who met the inclusion criteria to participate in the study. The following inclusion criteria were used:

- having HIV or AIDS
- English communication skills
- received their ART from either of the three public hospitals utilised for the research
- on treatment for not less than 6 months.

All of the participants received an explanation of the study’s goal. All participants were assured of their confidentiality and autonomy and invited to participate in the study. The data for this study were gathered through semi-structured interviews. The semi-structured interview guide was developed by the researchers and provided the participants with the opportunity to express their views. A pilot study was conducted in one of the hospitals selected for the study to test the validity of the interview guide within the context of the study. There was no amendment to the interview guide after the pilot study because it measured what the researchers intended to measure. Data analysis occurred simultaneously with data collection and stopped when data saturation was achieved, which occurred after the 15th semi-structured interview when no new data emerged. All interviews were recorded using a voice recorder, with the participants’ permission. The interviews were conducted in English because each participant’s ability to communicate in English was part of the inclusion criteria. The data were analysed using content analysis, employed using the six steps of data analysis prescribed by Creswell (2014). The first step involved the organisation and preparation of the data. This was followed by the second step, during which the transcribed data were scrutinised; thereafter, the data were coded, constituting the third step. The data were coded using Tesch’s eight steps in the coding process. Tesch’s eight steps are as follows: all the transcriptions were read carefully to get a sense of the whole.

Further analysis of each document was conducted to identify additional underlying meanings. This process was continued for all the other transcribed data as well as clustering those that were similar, being made. Codes were assigned to the topics. Categories were then assigned to the topics and grouped together as relevant; in addition, any interrelationships were observed. Each group was given a shorthand name. By combining the data for each group, a preliminary analysis was performed. The current data were recoded as necessary. The categories or themes were created from the analysis using the coding procedure, which also described the context of the participants. An impartial coder was employed to aid in the process of theme identification. Before the themes were finalised, the researcher, the independent coder, and the study promoters had several talks to reach consensus (fourth step). A description of how the themes and descriptions would be conveyed in a qualitative narrative was given (fifth step). Finally, an interpretation of the findings was made (sixth step). When discussing the themes, the researcher’s narratives were supported by the necessary verbatim quotations from the data they had gathered.

### Ethical considerations

A research proposal was approved by the Research Ethics Committee (human) of Nelson Mandela Metropolitan University (Now Nelson Mandela University) (H14-HEA-NUR-029), Institutional Review Board of 37 Military Hospital (37MH-IRB IPN 024/2015) and from the Ghana Health Service Ethics Review Committee (GHS-ERC:13/05/15). The participants were informed of their rights as participants. Participants who were willing to participate in the study and fit the inclusion criteria were given two consent forms to sign to keep a copy of the original signed form. The ethical principles of beneficence, respect for persons and justice were applied throughout the study. The researcher employed various strategies to ensure that a high ethical standard was maintained in the study, including maintaining privacy and confidentiality, informed consent, autonomy and avoiding discrimination. Trustworthiness was ensured using Lincoln and Guba’s framework, which comprised credibility, transferability, dependability, confirmability, and authenticity. Ethical principles such as beneficence and non-maleficence, respect for human dignity, justice, veracity, privacy, and confidentiality were considered in the study (Adler 2022).

## Results

The participants comprised seven males and eight females, with their ages ranging from 27 to 63 years. Their duration on ART ranged from 9 months to 11 years. From the data analysis, one main theme emerged with six sub-themes. The main theme that emerged was the diverse experiences expressed by the participants regarding their diagnosis and living with HIV/AIDS. The diverse experiences shared by the participants related to the emotions they felt when they first learned of their positive HIV status. The participants also shared experiences of stigmatisation and discrimination by their friends, neighbours, family, and partners. The participants shared their experiences regarding the adverse effects of the medication as well as those experiences related to the adherence challenges they faced. Furthermore, the participants expressed their lifestyle modification experiences and enhanced health condition when taking ARTs. The six sub-themes discussed below provide a detailed description of the diverse experiences expressed by the study participants.

### Sub-theme 1: Various emotions experienced after being diagnosed with HIV

The participants acknowledged feeling a range of emotions, such as shock, disbelief, sadness, depressed feelings, fear of death, and suicidal tendencies when they first received news of their positive HIV status. This was because some participants believed that being diagnosed with HIV was a sign of promiscuity, hence their diagnosis was unexpected because they thought they were not promiscuous. The participants reported being so stunned that it left them physically weak and, in some cases, prevented them from falling asleep for days:

‘… When I was told [*that I am HIV positive*] I was shocked. In fact, I thought all was gone because I had no idea. In fact, it was not easy for me at all because telling me that alone, I got weak, I got very weak, and I could not sleep for almost three days.’ (Participant 4, male, 40 years)

‘I was just shocked. Something was just going through me. I was not the type who jumps from one man to another …’ (Participant 1, female, 39 years)

A positive HIV diagnosis was difficult for some participants to accept, and they acknowledged their feelings of disbelief. To establish their HIV status, they even underwent a second test at a private pathologist. One married participant said she was shocked when she learned her husband had tested negative for HIV previously, so she expressed her shock at the news:

‘Actually, I did not believe it. It was … I was not scared though, but I did not believe it, so I had to go for a test in a private laboratory where no one asked me to go. I went there, and then, it was still positive …’ (Participant 14, female, 32 years).

‘… I felt surprised, I was surprised about it [*HIV positive result*] because as a married woman I was told my husband was not having the disease but I had … in fact, it was a surprise thing to me … very surprised.’ (Participant 7, female, 42 years)

Some participants indicated that they had shown suicidal thoughts when they were diagnosed as HIV positive:

‘… *when I left the hospital, on my way, just at the main gate, I met a man selling medicines for rats so I said this is the best way to end it for me; so, I bought it and joined the car* … *I alighted, then I saw this tipper truck coming … then I said oh this is an opportunity this can even make it faster so I stood in the middle of the road* … *so there was this guy that was also trying to cross the road so he came to pull me so we all went to the other side of the road* …’ (Participant 12, female, 42 years)

The participants had restless nights because they believed that HIV was a deadly disease that might easily claim the life of the patient at any time. The individuals were broken and distressed by their fear of dying. The extreme weight loss and physical changes they underwent, which are typical of people with HIV in its latter stages, brought them very close to death. Sadness and depression were triggered by these ideas and feelings:

‘… I felt very bad, very bad. I thought I would die because I became very lean …’ (Participant 6, male, 38 years)

‘I was very down, thinking I was going to die probably the next month or next two months to come, or a year. I was devastated and very much sad.’ (Participant 15, male, 50 years)

### Sub-theme 2: Experiences related to stigma and discrimination

The experience of stigmatisation was evident in one participant’s description of his home situation. The participant explained that he was not allowed to use the restroom or toilet facilities inside his family home because of his HIV-positive status. Another participant’s family resisted acknowledging her status and refused to live with her. The participants felt stigmatised as a result of their family member’s refusal to utilise facilities that the participants had used after they told them they were HIV positive out of concern that they may catch the virus themselves. The participants who tested positive for HIV had some family members who did not support them and did not want to interact with them. In one example, the family members rejected the food prepared by the participant because they had heard of her HIV-positive status from her aunt, a midwife who worked at the hospital where she was admitted. Before becoming aware of her HIV status, the family members enjoyed the food the participant cooked for the family. In addition, it was found that the children of HIV-positive persons also experienced discrimination. This was evident in family members being afraid to hold or touch the children of the study participants; they also prevented their children from playing with the children of HIV-positive family members. One participant describes her experience as follows:

‘I felt sick, I was admitted here and so an Auntie who was a Midwife came to see me and she saw it in my folder. She went to the extent of telling my cousins and others in the house. … I sometimes cook for all in the house to eat and, when I cook, nobody will eat again … and even my child, nobody will even touch him or even play with the child.’ (Participant 12, female, 42 years)

Participants disclosed that their husbands had mistreated them. One participant claimed that her spouse did not feel the need to stay by him and provide him with the required love and support because she saw him as useless and someone who would soon pass away. Another participant said his wife was unkind, prejudiced, and unprepared to care for him. Because of the husband’s positive HIV test, the wife lost interest in the marriage; she demanded that he move in with his parents so that they could take care of him, and she threatened to file for divorce. The participant said he was upset by the wife’s conduct and felt abandoned by her. Thus, he wanted God to punish her. He passionately shared the following account of his experience:

‘I was sick and my wife called my parents and told them that if they do not come for me, and I die, they should not ask her anything. My wife says she is divorcing me … she told me you are sick why don’t you resign from your work, pack your things and go to the village. I mean, if God is a person like you, I will tell Him to punish her seriously …’ (Participant 6, male, 38 years)

As a result of their HIV-positive status, the participants reported experiencing varying degrees of stigmatisation and prejudice from friends and neighbours. They largely refrained from sharing anything personal with the participants, which clarified this. When a friend he formally shared the same bath towel with said he did not want him to use his bath towel because he was HIV-positive, one participant felt discriminated against. Another participant felt discriminated against when she was not allowed to use her own bucket to gather water from the household’s shared tap because the other members believed she and her personal possessions were infected and would spread the infection to them. These experiences of discrimination and stigmatisation are described as follows:

‘… I was using someone towel but then I had left my place and I was residing somewhere when I was discharged from the hospital, and the person complained …’ (Participant 8, male, 60 years)

‘There was this advert for HIV ambassadors; I was also one of them. So, the day I came out, they saw that thing on television and people were like “hey …” Even I rented a single room that I was staying in with the child then my landlord, I think they saw it then they started. That day, I went home and I wanted to fetch water, she shouted and said “do not fetch madam, move away”. Then I was like what is it? Nobody in the house talks to me …’ (Participant 12, female, 42 years)

Because they had not disclosed their status, some participants acknowledged that they had not encountered any stigmatisation or discrimination. It was a prerequisite for the start of ART that one disclose his or her status to at least one person who could act as a treatment supporter; therefore, some were forced to do so. Some participants in this study felt comfortable keeping their status a private and personal secret because they were not comfortable disclosing their status to fellow employees, parents, siblings, and friends. These are reflected in the following quotes:

‘Nobody knows in the work side nobody knows …’ (Participant 6, male, 38 years)

‘I do come to the hospital with my mum; but she doesn’t know my status …’ (Participant 14, female, 32 years)

‘… I haven’t told anyone of my status apart from my family. I do not believe in telling people, that is one thing about me. With my job you go to people’s home and I hear them talk about it and the way a manner they act is like hey you have to be very careful …’ (Participant 1, female, 39 years)

### Sub-theme 3: Side-effects related to ART

A challenge that several participants emphasised was the adverse effects that the participants encountered while using ART. The individuals reported adverse effects such as headaches, nausea, vomiting, nightmares, diarrhoea, and an overall feeling of being unwell. Some participants said that the counsellor had informed them of any potential drug side effects, while others insisted that they had not been told what to expect regarding adverse effects. Some of the participants thought that some of the medications used as part of the treatment had side effects that were quite noticeable. This was expressed by one participant as follows:

‘… the counsellor told me about the side-effects that you can have, some rashes, and really too I had it … and some dreams and the dizziness also. The experience I had when I started taking it, it was … uhm … you know … like a nightmare, some strange dreams always feeling very dizzy. The dizziness was very serious …’ (Participant 8, male, 60 years)

‘… the medication they did not tell me it will [*have an effect on me*] … when I started initially like for the start I will have some symptoms like dizziness. Vomiting etc with my story when I started I had this nightmare seriously …’ (Participant 2, female, 40 years)

‘… some of them their eye, everybody and the reaction but me I didn’t see any reaction on it only the toilet for three days … we have problem with it at times they give you some the specific one if you take it you feel fine your body will be fine at times; if they give you some you do not feel fine your body will not be fine.’ (Participant 11, male, 32 years)

### Sub-theme 4: Managing HIV requires a change in lifestyle

The participants said that to manage their positive HIV status and to keep their health, they had to change their way of living. According to the following quotes, the participants’ lifestyles changed to include giving up alcohol, altering their diets, taking dietary supplements, consuming fewer sugars, exercising, and ceasing to engage in immoral sexual behaviour:

‘… it is that now I take very good care of myself. I do not do things that will disturb me. I do not eat anything, I do not do things that will disturb my life, I do not go out with friends drinking and doing all sort of immoral things. So, when I close from work … straight away, I came to the house.’ (Participant 3, male, 51 years)

The participants admitted that it took them a while to accept that they would need to take medication regularly for the rest of their lives. They have since grown accustomed to this idea, and they also seem at ease and to be managing taking their prescription on a regular basis. One participant responded as follows:

‘You know, it was difficult because – for me – I do not like taking medicine. So, when I was told I was going to be on it for life I said “wow” … it was not easy; but, now, I will say I think it is part of me now.’ (Participant 15, male, 50 years)

### Sub-theme 5: Improved health on ART

The participants said that after starting ART, their health has improved. Some participants reported that, in contrast to their prior experiences when they experienced high fevers, they only encountered moderate fevers after beginning ART. Some of the participants claimed that the ART boosts their immune system, while others stated that the ART made them strong enough to stand up and take care of themselves without feeling helpless. One participant noticed the following:

‘Madam, since I was put on this drug, I have never been sick that I should say I am sick … maybe minor cold, fever … not really a fever, when I remember when I wasn’t on this drug, I could get a severe fever … my medication is making me look good and good and good and good. Up to now, I think the medication is really doing me good, it is keeping me going.’ (Participant 4, male, 40 years)

The participants’ better adherence to the treatment was motivated by the health improvements they noticed while receiving their ART:

‘… in fact at the initial stage I was weak and since I started taking it I got up and later I could do everything so I know that can help me …’ (Participant 9, male, 63 years)

### Sub-theme 6: Barriers to treatment

As a result of certain barriers, the participants found it challenging to follow ART recommendations. The study participants showed a desire to learn more about HIV and believed that if they knew more about it, they would respond to the drug more successfully. The following statement illustrates this:

‘If more education is given to me, I will be ok with it. I want to know about it … I want to know more about the HIV.’ (Participant 15, male, 50 years)

Another barrier noticed in the following statement made by a participant as contributing to their main cause of non-adherence to the treatment was drug forgetfulness:

‘Once in a while you may go somewhere, or you may be in a haste to go somewhere, you forget to take your drug … so you can miss it in the morning, but in the evening you take.’ (Participant 10, female, 32 years)

According to the participants, one of the biggest obstacles to sticking with the treatment is travelling and leaving the medication behind. An unpredictable length of stay when they travel was a shared challenge, as observed in the following statement:

‘I think it was once I travelled at a weekend and I didn’t take it for … I think … two days. In fact, I did not know I was going to travel when I came to work before I was asked to travel, so that, I didn’t take it. I didn’t bother though. When I came back I reported to the doctor, and he said there is no problem with that so I didn’t have any problem.’ (Participant 9, male, 63 years)

Financial difficulty was another barrier to ART adherence, as the participants shared. The inability to pay for transportation to the ART clinic to pick up their antiretroviral therapy is one financial hurdle to adherence, as is the lack of money to buy food that the body needs before taking the medications and the inability to pay GHS 5.00 for the highly subsidised ART. One participant highlighted the barriers he faced as follows:

‘We buy the drugs at GHS 5; so, if they can make it free, it will help, because somebody do not have. They do not have money, that is why they do not come … if they do not have money they will not go and people do not take because it will finish. So, if you can tell them that the drug should be free …’ (Participant 5, male, 48 years)

## Discussion

The participants in this study acknowledged that they felt a range of emotions, such as shock, disbelief, sadness, depressed feelings, fear of death, and suicidal tendencies, when they first received news of their positive HIV status. The findings of this study are consistent with those of Arias-Colmenero et al. (2020), who reported that diagnosis of HIV resulted in feelings such as disappointment, sadness, fear, and despair. Similar studies by Owusu (2022) on experiences of new diagnoses among HIV-positive persons are as follows: implications for public health reported the overwhelming majority of respondents reported a wide range of unfavourable psychosocial reactions, including suicidal ideation, following their new HIV-positive infection diagnosis. Horter et al. (2017) also documented that after learning they had an HIV infection, several participants said they were shocked, upset, and confused about how they got the virus and where it came from. Several people had trouble accepting the diagnosis; some had doubts or refused to believe the test results.

Participants from this study experienced stigmatisation and discrimination from their spouses, siblings, and friends. They faced rejection, a lack of support, and the threat of divorce. Fear of discrimination and stigmatisation discouraged some participants from disclosing their HIV-positive status. These are likened to the experiences described by the participants in other studies, which found that people frequently maintained distance from HIV-positive individuals. Some participants experienced marriage breakdowns, unemployment, and social isolation and could not perform everyday chores and responsibilities. The leading cause of the hesitation to report HIV-positive status has been fear of stigmatisation and prejudice (Opoku et al. 2021; Sariah et al. 2016). These results, however, differ from those of a study conducted by Patel et al. (2012), which found that there was a high disclosure rate among their participants who experienced improved self-esteem, fewer symptoms, and better quality of life because they were able to disclose their HIV positive status to someone. The experiences of participants in this study are similar to the findings from the study on PLHIV stigma index study in Ghana, which found that the worst forms of stigma experienced by the respondents were gossip and verbal insults or harassment, which featured an average of 63% and 79%, respectively. Also, the PLHIV respondents avoided all forms of social exclusion and other forms of discrimination through non-disclosure of their HIV status (Stigma Index 2014).

The side effects of ART experienced by the participants in this study were similar to Agu and Oparah (2013), who found that 47.7% of the participants in their study also reported adverse drug reactions after initiating ART. In a related study in India conducted by Shet et al. (2014), 90% of 321 participants experienced at least one adverse drug reaction. However, it was established that a higher degree of adverse drug reactions occurs among clients on first-line ART (Shet et al. 2014). The participants in this study indicated that they had to adapt to a new lifestyle because HIV is a lifelong chronic condition that requires prolong treatment. According to Bezabhe et al. (2014) patients‘ better health motivated them to continue taking their medication. The improved health of the participants in this study was evident in their experiences of improving their immune system, as they noticed that they were less prone to developing any illnesses since being on ART.

Among the barriers that the study found included a desire to learn more about HIV itself, drug forgetfulness, travelling and leaving the medication behind, financial difficulty, inability to pay for transportation to the ART clinic to pick up their antiretroviral therapy and the lack of money to buy food that the body needs before taking the medications. Various other studies highlighted similar barriers to those identified in this study. Muduka and Tobin-West (2015) reported that non-adherence to the recommended course of therapy is influenced by a lack of awareness of how ART medications work. According to Oku et al. (2013), being very busy and forgetting to take the medication was the main reason for missing a dosage of ART. According to Taylor et al. (2014), a barrier to treatment compliance for clients who live far from ART clinics is the increased cost of transportation. Similar research by Musumaria et al. (2013) revealed that patients reported having trouble finding the money to pay for transportation to the relevant clinic.

Among the strengths of the method used in this study is the ability to answer questions that are subjective to the participants. However, this limits the generalisation of the findings. Another limitation to this study is only using public hospitals, hence the findings are particularly related to those facilities. The privacy associated with HIV/AIDS management and care limits the openness of participants to share their experiences.

## Conclusion

From the aforementioned discussion it is evident that people living with HIV/AIDS have diverse experiences related to their emotions and other aspects consequent to their diagnosis. Significant effort should therefore be made to accept people living with HIV and provide them with the necessary support. The data analysis made it evident that stigmatisation and prejudice played a role in patients‘ non-adherence to ART. It is advised that more investigation be carried out into the effects of stigma and discrimination on achieving the best possible adherence to ART. There should also be educational sessions and workshops on HIV and ART in order to enhance patient education so that patients on ART are provided with the most important information on the condition and the medication. It is worth noting that the experiences of people living with HIV/AIDS in the context of this study are similar to the experiences of others living in similar conditions elsewhere in the world.
